# Increased expression of miR-27 predicts poor prognosis and promotes tumorigenesis in human multiple myeloma

**DOI:** 10.1042/BSR20182502

**Published:** 2019-04-09

**Authors:** Feifei Che, Chunqian Wan, Jingying Dai, Jiao Chen

**Affiliations:** Department of Hematology, Sichuan Academy of Medical Science and Sichuan People’s Hospital, Chengdu 610072, China

**Keywords:** cell invasion, miR-27, multiple myeloma, poor prognosis, SPRY2

## Abstract

Multiple myeloma (MM) is an incurable hematological malignancy characterized by abnormal infiltration of plasma cells in the bone marrow. MicroRNAs (miRNAs) have emerged as crucial regulators in human tumorigenesis and tumor progression. miR-27, a novel cancer-related miRNA, has been confirmed to be implicated in multiple types of human tumors; however, its biological role in MM remains largely unknown. The present study aimed to characterize the biological role of miR-27 in MM and elucidate the potential molecular mechanisms. Here we found that miR-27 was significantly up-regulated in MM samples compared with normal bone marrow samples from healthy donors. Moreover, the log-rank test and Kaplan–Meier survival analysis displayed that MM patients with high miR-27 expression experienced a significantly shorter overall survival than those with low miR-27 expression. In the current study, we transfected MM cells with miR-27 mimics or miR-27 inhibitor to manipulate its expression. Functional studies demonstrated that miR-27 overexpression promoted MM cell proliferation, facilitated cell cycle progression, and expedited cell migration and invasion; whereas miR-27 knockdown inhibited cell proliferation, induced cell cycle arrest, and slowed down cell motility. Mechanistic studies revealed that Sprouty homolog 2 (SPRY2) was a direct target of miR-27 and that rescuing SPRY2 expression reversed the promoting effects of miR-27 on MM cell proliferation, migration, and invasion. Besides, miR-27 ablation suppressed tumorigenecity of MM cells in mouse xenograft models. Collectively, our data indicate that miR-27 exerts its oncogenic functions in MM by targetting SPRY2 and that miR-27 may be used as a promising candidate target in MM treatment.

## Introduction

Multiple myeloma (MM), the second most frequent human hematological malignancy, is characterized by abnormal filtration of clonal plasma cells in the bone marrow [1,2]. MM accounts for nearly 1% of human cancers worldwide, with approximately 74000 new cases occurring annually [3]. As estimated by the American Cancer Society, approximately 28850 new MM cases were diagnosed in the United States in 2015, and more than 11000 patients died of this malignancy [4]. It is well acknowledged that uncontrolled cell growth and malignant metastasis may be partially responsible for the high mortality of MM patients [5,6]. Despite the fact that significant progress has been achieved in the diagnosis and treatment of MM, long-term prognosis of patients remains rather poor. Thus, it is imperative to develop novel and effective therapeutic strategies for MM.

MicroRNAs (miRNAs) are a large group of small single-stranded, non-coding RNA molecules (typically ∼22 nts in length) that negatively regulate gene expression at post-transcriptional level by binding to the 3′UTRs of their target mRNAs [7,8]. Accumulating evidence has demonstrated that aberrant expression of miRNAs play critical roles in the initiation and malignant progression of human tumors [9,10]. miR-27, a novel cancer-related miRNA, has been identified to be implicated in several types of human malignancies, including gastric cancer [11], breast cancer [12], esophageal cancer [13], and thyroid cancer [14]. Nonetheless, the biological role of miR-27 in human MM remains poorly understood. The purpose of the present study was to characterize the biological role of miR-27 in MM and clarify the potential molecular mechanisms involved.

A growing number of studies have reported that miRNAs exert their biological functions in human MM by modulating target gene expression [15–17]. Furthermore, it is of vital importance to reveal the close association between miRNAs and their target genes for illuminating the potential molecular mechanisms underlying MM occurrence and progression. It is well documented that the Sprouty (SPRY) family of proteins are a highly conserved group of negative feedback loop modulators of mitogen-activated protein kinase (MAPK) signaling pathway [18]. SPRY homolog 2 (SPRY2) belongs to the SPRY family of proteins. Previous studies have demonstrated that SPRY2 acts as a tumor suppressor in diverse types of human cancers, such as non-small cell lung cancer [19], liver cancer [20], colon cancer [21], and breast cancer [22]. Moreover, SPRY2 has also been reported to be a critical regulator in MM and exert its tumor-suppressing roles by inhibiting cell proliferation, survival, and metastasis [23,24].

In the current study, we found that miR-27 was significantly up-regulated in MM tissues compared with normal bone marrow tissues of healthy donors. Functional studies showed that miR-27 facilitates MM cell proliferation, cell cycle progression, migration, and invasion. Furthermore, mechanistic investigations revealed that miR-27 exerted its promoting effects on MM cell proliferation and motility by directly targetting SPRY2.

## Materials and methods

### Patients and tissue samples

Bone marrows were collected from 60 MM patients and 60 healthy volunteers at Sichuan Academy of Medical Science and Sichuan People’s Hospital (Chengdu, China) between February 2008 and December 2016. Clinicopathological parameters of MM patients were listed in Table 1. Overall survival time was defined as the interval between the date of primary surgery treatment and the date of death or last follow-up. All the patients gave their written consents. The present study was approved by the Ethics Committee of Sichuan Academy of Medical Science and Sichuan People’s Hospital. The present study was conducted in accordance with the principles of Declaration of Helsinki.

**Table 1:** Correlation between miR-27 expression and clinicopathological characteristics of 60 MM patients

Features	Number of cases	miR-27 expression		*P*-value
		High (*n*=34)	Low (*n*=26)	
Age (years)				0.602
<55	30	18	12	
≥55	30	16	14	
Gender				0.558
Female	29	15	14	
Male	31	19	12	
IMWG risk				0.008
Low risk	14	3	11	
Intermediate risk	18	11	7	
High risk	28	20	8	
ISS stage				0.025
I	15	4	11	
II	19	13	6	
III	26	17	9	
ASCT treatment				0.463
Performed	27	14	13	
Not performed	33	20	13	
Plasma cell morphology				0.628
Plasmacytic	18	9	9	
Plasmablastic	22	12	10	
Mixed	20	13	7	

Abbreviations: ASCT, autologous stem cell transplantation; IMWG, International Myeloma Working Group; ISS, International Staging System.

### Cell lines and cell culture

Normal plasma cells (NPCs) and four MM cell lines (U266, RPMI8226, MM.1S, and H929) were purchased from Shanghai Cell Bank of Chinese Academy of Sciences (Shanghai, China). All the cells were cultured in RPMI-1640 medium (Gibco, Grand Island, NY, U.S.A.) supplemented with 10% FBS (Gibco). The cells were maintained at 37°C in a humidified atmosphere with 5% CO_2_.

### Cell transfection

Cell transfection was carried out using Lipofectamine 2000 (Invitrogen Life Technologies, Carlsbad, CA, U.S.A.) according to the manufacturer’s protocol. MiR-27 mimics and miR-27 inhibitor were designed and synthesized by GenePharma Co. Ltd (Shanghai, China). Sequence of miR-27 mimics was listed as follows: 5′-UUCACAGUGGCUAAGUUCCGC-3′. The cells were harvested for further studies at 48 h post-transfection. The transfection efficiency was evaluated by quantitative real-time PCR (qRT-PCR).

### Cell proliferation assays

Cell proliferation was evaluated using MTT Cell Proliferation Assay Kit (Thermo Fisher Scientific, San Jose, CA, U.S.A.) following the manufacturer’s protocol. Briefly, 20 μl MTT solution (Merck Millipore, Billerica, MA, U.S.A.) at a concentration of 5 mg/ml was added to each well of 96-well culture plate and incubated for another 4 h at 37°C. The supernatants were discarded, and 150 μl DMSO was added to the cells. The absorbance was measured at 570 nm using a microplate reader (Bio-Tek Instruments, Winooski, VT, U.S.A.). All the experiments were performed in triplicate.

### Cell cycle analysis

For cell cycle analysis, cells were trypsinized using 0.25% trypsin, washed twice with PBS, and then fixed in 70% ethanol at 4°C overnight. The cells were then stained with 10 μl propidium (PI, Sigma–Aldrich, U.S.A.) that contained 10 μg RNase A (Sigma–Aldrich) for 30 min at 4°C in the dark. Cells were analyzed using a flow cytometer (Becton-Dickinson, San Jose, CA, U.S.A.).

### Cell apoptosis analysis

Cell apoptosis was evaluated using an Annexin V-FITC Apoptosis Detection Kit (BD Biosciences; San Jose, CA, U.S.A.) following the manufacturer’s protocols. Briefly, cells were seeded in six-well plates at a concentration of 10^5^/ml and allowed to grow for 24 h. Then the cells were collected, washed three times with cold PBS, and re-suspended in 1× binding buffer. Subsequently, the cells were incubated with Annexin V-FITC/PI for 15 min in the dark at room temperature. Finally, the cells were analyzed using a flow cytometer (Becton Dickinson).

### Wound healing assays

MM cell migration ability was assessed using wound healing assays as previously described [25]. In brief, an artificial wound was produced using a 200-μl pipette tip on the confluent cell monolayer. Following further incubation in the culture medium for 24 h, closure of scratches was observed using an inverted microscope (Olympus, Tokyo, Japan). Cells were observed and photographed at 0 and 24 h after wounding under an inverted light microscope (Olympus) at 100× magnification, respectively. The cell-free area at 24 h after scratching and the original denuded area were measured using the ImageJ software (National Institute of Health, Bethesda, MD, U.S.A.).

### Transwell invasion assays

MM cell invasion capability was determined by Transwell invasion assays using 24-well transwells (8 μm pore size; Millipore) coated with Matrigel (BD Bioscience, San Jose, CA, U.S.A.) according to the manufacturer’s protocol. In brief, approximately 1 × 10^4^ cells were seeded into the upper chambers containing FBS-free DMEM. The lower chambers were filled with DMEM containing 10% FBS. The Transwell plates were then incubated for 24 h. Then cells that migrated through the membrane were fixed in 4% paraformaldehyde and stained with 0.5% Crystal Violet (Sigma) for 30 min. Then the migrated cells from five random visual fields were counted under a light microscope (Nikon, Tokyo, Japan) and photographed at a magnification of 400×.

### Western blotting analysis

Total proteins were extracted using RIPA buffer supplemented with protease inhibitor cocktail (Roche, Basel, Switzerland). The protein samples were separated using SDS/PAGE and then transferred on to the PVDF membranes. After being blocked with 5% degrease milk in TBST buffer, the membranes were incubated with primary antibodies overnight at 4°C. Anti-SPRY2 (ab85670) and anti-GAPDH (ab37168) were purchased from Abcam (Cambridge, MA, U.S.A.). Antibodies were used at the following dilutions: anti-SPRY2 (1:1000) and anti-GAPDH (1:2000). After being washed three times, the membranes were then incubated with horseradish peroxidase (HRP)-labeled secondary antibody at 37°C for 1 h. The blots were developed using the ECL Western Blotting Detection Kit (Amersham, Buckinghamshire, England).

### qRT-PCR

Total RNA was extracted using TRIzol Reagent in accordance with manufacturer’s instructions. For miRNA expression analysis, miR-27 was reverse-transcribed using TaqMan MiRNA Reverse Transcription Kit (Applied Biosystems, Foster, CA, U.S.A.). U6 was used as an endogenous control to normalize miR-27 expression. For *SPRY2* mRNA expression, the first stand was synthesized using TaqMan High-Capacity cDNA Reverse Transcription Kit (Applied Biosystems). GAPDH was used as the internal control for normalization of *SPRY2* mRNA expression. PCR was performed on an Applied Biosystems 7500 Fast Real-time PCR system. The sequences of specific primers were listed as follows: miR-27, forward 5′-CGCCTTGAATCGGTGACACTT-3′ and reverse 5′-GGCAAGTGTCACCGATTCAAG-3′; SPRY2, forward 5′-CTAAGCCTGCTGGAGTGACCG-3′ and reverse GTGTTTCGGATGGCTCTGATG; GAPDH, forward 5′-CACCATCTTCCAGGACGAG-3′ and reverse 5′-CCTTCTCCATGGTGGTGAAGA-3′; U6, forward 5′-GCTTCGGCAGCACATATACTAT-3′ and reverse 5′-CGCTTCACGAATTTGCGTGCAT-3′. The relative transcript abundance was determined according to the 2^−ΔΔ*C*^_t_ method. All the experiments were carried out in triplicate.

### Luciferase reporter assays

Luciferase reporter assays were used to validate the direct binding of miR-27 and SPRY2 mRNA 3′UTR fragments as previously described [26]. In brief, wild-type and mutant SPRY2 mRNA 3′UTR fragments were cloned into the pGL3-basic dual luciferase reporter vectors (Promega, Madison, WI, U.S.A.), respectively. A dual-luciferase reporter system (BD Bioscience) was used to determine the relative luciferase activity at 48 h post-transfection. Firefly luciferase activity was normalized to *Renilla* luciferase activity.

### Tumorigenicity in nude mice

Male BALB/c nude mice (5–6 weeks of age, *n*=12) were purchased from Guangdong Medical Laboratory Center (Guangzhou, China) for the establishment of the subcutaneous xenograft tumor models. Briefly, negative control (NC) mimics or miR-27 mimics-treated MM cells (5 × 10^6^ cells per mouse) were subcutaneously injected into the flanks of the nude mice. Tumor size was measured using slide caliper every 3 days, and tumor volumes were calculated according to the formula: volume (mm^3^) = [width^2^ (mm^2^) × length (mm)]/2. At day 21 post-inoculation, all the nude mice were killed, and the tumors were collected and weighted. This animal protocol was approved by Animal Care and Use Committee of Xi’an Jiaotong University (Xi’an, China).

### Statistical analysis

Data were presented as mean ± S.D. Statistical analysis was conducted using SPSS 18.0 software (SPSS Inc., Chicago, IL, U.S.A.). Student’s *t* test was used to compare the difference between two groups. One-way ANOVA followed by the Dunnett’s multiple comparisons was applied to analyze the differences amongst three independent groups. Fisher’s exact test was employed to evaluate the relationship between miR-27 expression and clinicopathological characteristics of patients. *P*<0.05 was considered statistically significant.

## Results

### Increased miR-27 expression predicts poor prognosis of MM patients

**Figure 1:**
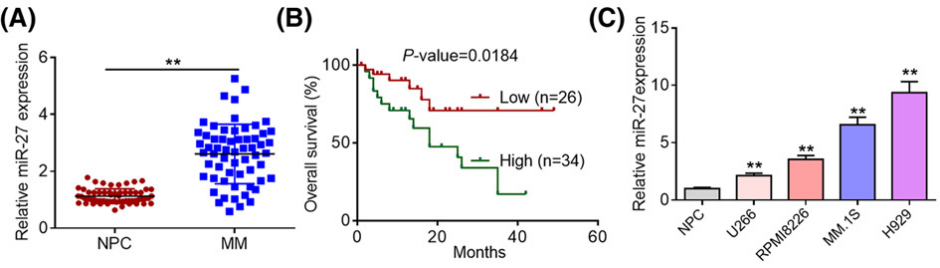
Increased miR-27 expression predicts poor prognosis of MM patients (**A**) Relative miR-27 expression levels in MM tissues of 60 patients and normal bone marrow tissues of 60 healthy donors were determined by qRT-PCR analysis. (**B**) MM tissues were divided into high miR-27 group and low miR-27 group based on the average value of its expression levels. The log-rank test and Kaplan–Meier survival analysis were conducted to evaluate the relationship between miR-27 expression and overall survival of MM patients. (**C**) Relative miR-27 expression levels in NPCs and four MM cell lines (U266, RPMI8226, MM.1S, and H929) were examined by qRT-PCR analysis. **P<0.01.

Although previous studies have identified miR-27 as a critical regulator in several types of human malignant tumors, its role in MM remains unclear. To explore the biological role of miR-27 in MM, we first conducted qRT-PCR assays to determine its expression patterns in MM samples of 58 patients and normal bone marrow tissue samples of 58 healthy donors. As presented in Figure 1A, MM tissues displayed higher miR-27 expression levels than normal bone marrow samples of healthy donors. To evaluate the correlation between miR-27 expression and clinicopathological characteristics of patients, MM tissues were classified into high miR-27 expression group and low miR-27 expression group based on the average value of its expression levels. Fisher’s exact test showed that increased expression of miR-27 correlated with high International Myeloma Working Group (IMWG) risk and advanced International Staging System (ISS) stage (Table 1). Furthermore, the log-rank test and Kaplan–Meier survival analysis demonstrated that patients with high miR-27 expression had a significantly shorter overall survival than those with low miR-27 expression (Figure 1B). Consistently, we observed that miR-27 was significantly up-regulated in four MM cell lines (U266, RPMI8226, MM.1S, and H929) compared with NPCs (Figure 1C). Taken together, our findings indicate that increased miR-27 expression predicts poor prognosis in MM patients.

### miR-27 facilitates MM cell proliferation, survival, and invasion

**Figure 2:**
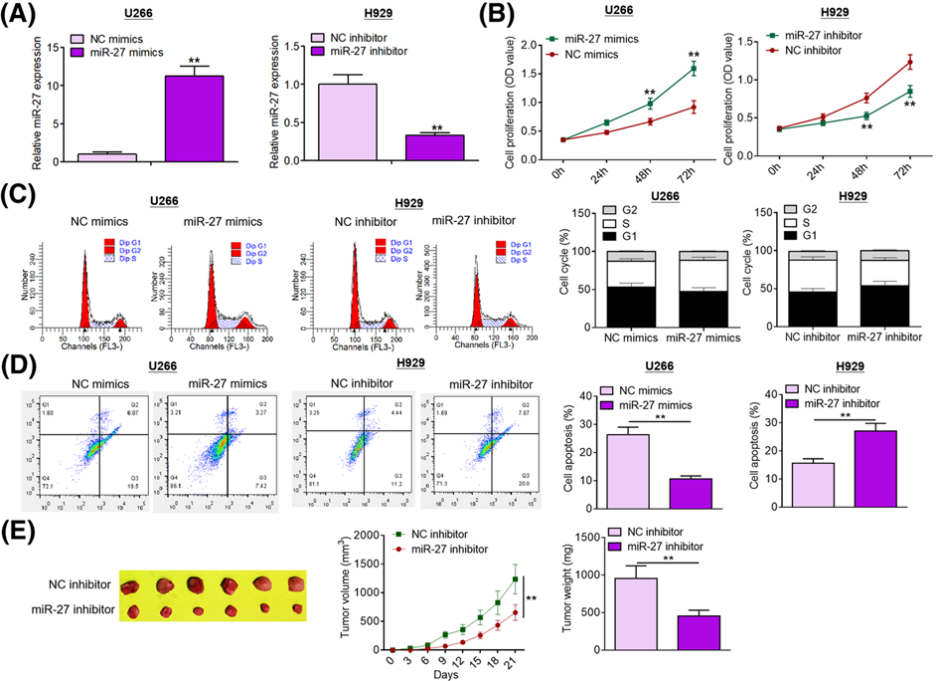
miR-27 facilitates MM cell proliferation and inhibited apoptosis (**A**) Transfection efficiency was assessed by qRT-PCR analysis. (**B**) Cell proliferation was evaluated by MTT assays after transfection with miR-27 mimics or miR-27 inhibitor. (**C**) Cell cycle was assessed by FCM after transfection with miR-27 mimics or miR-27 inhibitor. (**D**) Cell apoptosis was evaluated using FCM analysis after transfection with miR-27 mimics or miR-27 inhibitor. (**E**) miR-27 inhibitor-treated H929 cells were subcutaneously injected into the flanks of nude mice (*n*=6). Tumor size was determined by slide caliper every 3 days. At day 21 post-transplantation, nude mice were killed and tumors were weighed. **P<0.01.

**Figure 3:**
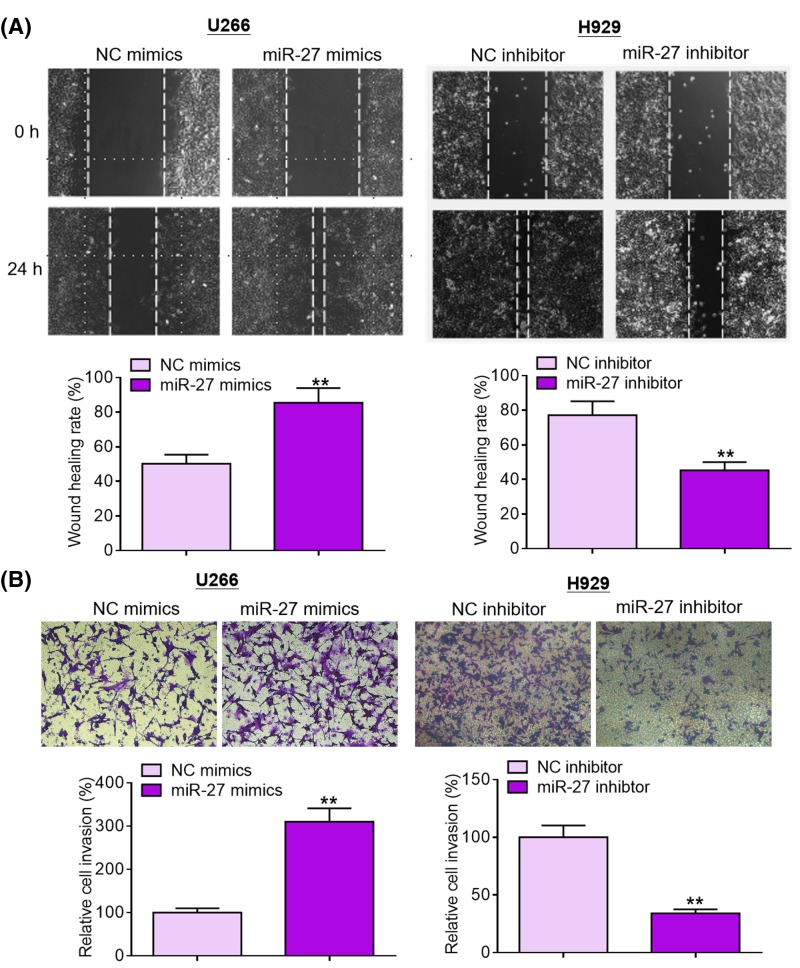
miR-27 promotes MM cell migration and invasion (**A**) Cell migration was examined by Transwell migration assays after transfection with miR-27 mimics or miR-27 inhibitor. (**B**) Cell invasion was detected by Transwell invasion assays after transfection with miR-27 mimics or miR-27 inhibitor. **P<0.01.

Given the data mentioned above, we speculated that miR-27 was involved in MM development and progression. Gain- and loss-of-function approaches were adopted to conduct functional investigations. In the current study, we transfected MM cells with miR-27 mimics or miR-27 inhibitor to manipulate its expression. Overexpression and knockdown studies were carried out in U266 cells (lowest endogenous miR-27 expression) and H929 cells (highest endogenous miR-27 expression), respectively. Transfection efficiency was determined by qRT-PCR analysis (Figure 2A). As shown in Figure 2B, miR-27 overexpression significantly promoted U266 cell proliferation compared with NC treatment, whereas miR-27 ablation dramatically suppressed H929 cell proliferation. As evident from flow cytometry (FCM) analysis, miR-27 overexpression accelerated U266 cell cycle progression compared with NC group, while miR-27 knockdown retarded H929 cell cycle progression (Figure 2C). As shown in Figure 2D, miR-27 overexpression inhibited U266 cell apoptosis compared with NC group, whereas miR-27 depletion promoted H929 cell apoptosis. To validate the effect of miR-27 *in vivo*, we established mouse xenograft models by subcutaneous injection of NC inhibitor or miR-27 inhibitor-treated H929 cells. As exhibited in Figure 2E, tumors harvested from miR-27 inhibitor group grew remarkably slower than those from NC group; moreover, tumors formed by miR-27 inhibitor-treated H929 cells weighed significantly lighter than those formed by NC inhibitor-treated H929 cells. Cell migration and invasion are two crucial events in the malignant progression of tumors. As displayed in Figure 3A,B, miR-27 overexpression accelerated U266 cell migration and invasion in comparison with NC treatment, whereas miR-27 depletion weakened the migration and invasion capabilities of H929 cells. Taken together, these results suggest that miR-27 promotes MM cell proliferation, survival, and motility.

### SPRY2 is a direct target of miR-27 in MM cells

**Figure 4:**
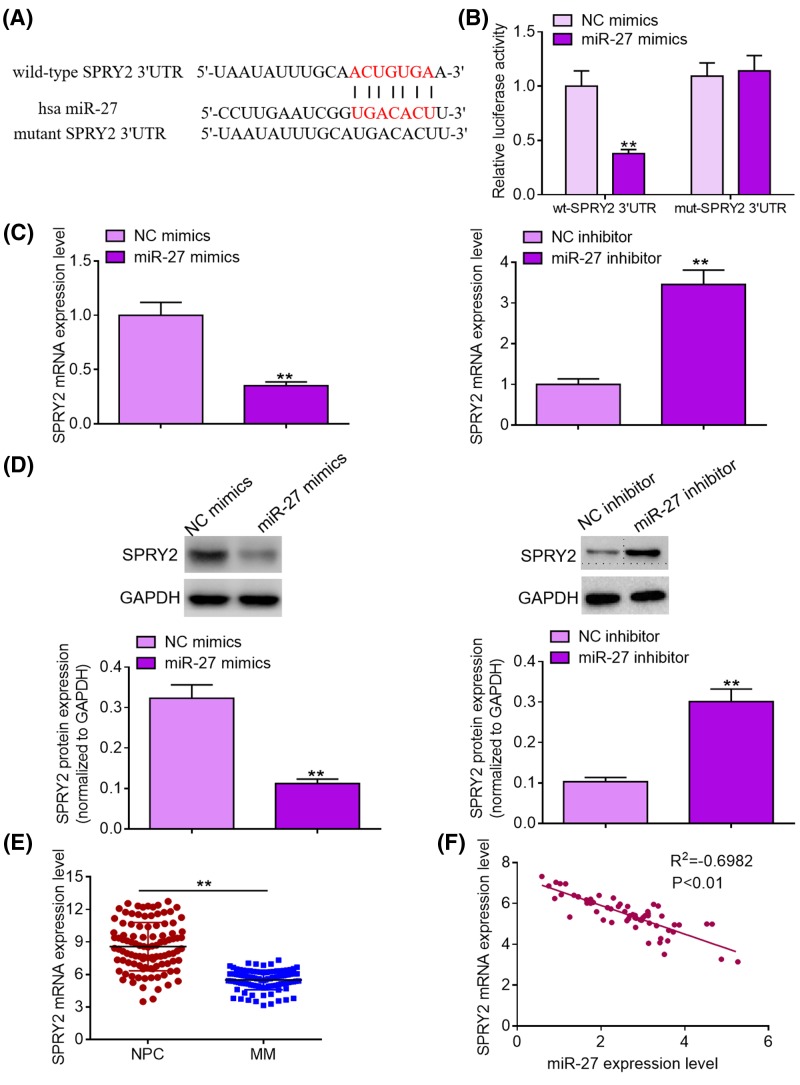
SPRY2 is a direct target of miR-27 in MM cells (**A**) A putative binding site of miR-27 in the 3′UTR of SPRY2 was predicted by TargetScan online software. (**B**) Luciferase activity of the reporter vectors carrying wild-type or mutant *SPRY2* mRNA 3′UTR fragments was examined after transfection with NC mimics or miR-27 mimics. (**C**) *SPRY2* mRNA expression was detected by qRT-PCR analysis after transfection with miR-27 mimics or miR-27 inhibitor. (**D**) SPRY2 protein expression was analyzed by Western blotting after transfection with miR-27 mimics or miR-27 inhibitor. (**E**) *SPRY2* mRNA expression levels in MM tissues of 60 patients and normal bone marrow tissues of 60 healthy donors were determined by qRT-PCR analysis. (**F**) Pearson’s correlation analysis was carried out to evaluate the relationship between miR-27 expression and *SPRY2* mRNA expression in MM tissue samples. **P<0.01.

To illuminate the potential molecular mechanisms by which miR-27 promotes MM cell proliferation, migration, and invasion, we conducted bioinformatics analysis using TargetScan online software. Amongst all the putative targets, SPRY2 drew our attention for its crucial role in multiple types of human tumors and was selected as a direct target of miR-27 (Figure 4A). Subsequently, we carried out dual luciferase reporter assays to verify whether miR-27 could binds to *SPRY2* mRNA 3′UTR. As exhibited in Figure 4B, transfection of miR-37 mimics significantly reduced the luciferase activity of the reporter vectors carrying wild-type SPRY2 mRNA 3′UTR fragments compared with NC treatment, whereas transfection of miR-27 mimics failed to trigger significant changes in the luciferase activity of the reporter vectors carrying mutant *SPRY2* mRNA 3′UTR fragments. Moreover, qRT-PCR analysis and Western blotting demonstrated that miR-27 overexpression significantly decreased the expression levels of *SPRY2* mRNA and protein compared with NC group, while miR-27 depletion dramatically increased the expression levels of *SPRY2* mRNA and protein (Figure 4C,D). Notably, we found that MM tissues displayed lower *SPRY2* mRNA expression levels than normal bone marrow tissues of healthy donors (Figure 4E). Besides, Pearson’s correlation analysis showed that miR-27 expression was inversely correlated with *SPRY2* mRNA expression in MM tissues (Figure 4F). To sum up, our data indicate that SPRY2 is a downstream direct target of miR-27 in MM cells.

### Rescue of SPRY2 expression reverses the promoting effects of miR-27 on MM cell proliferation, survival, and invasion

**Figure 5:**
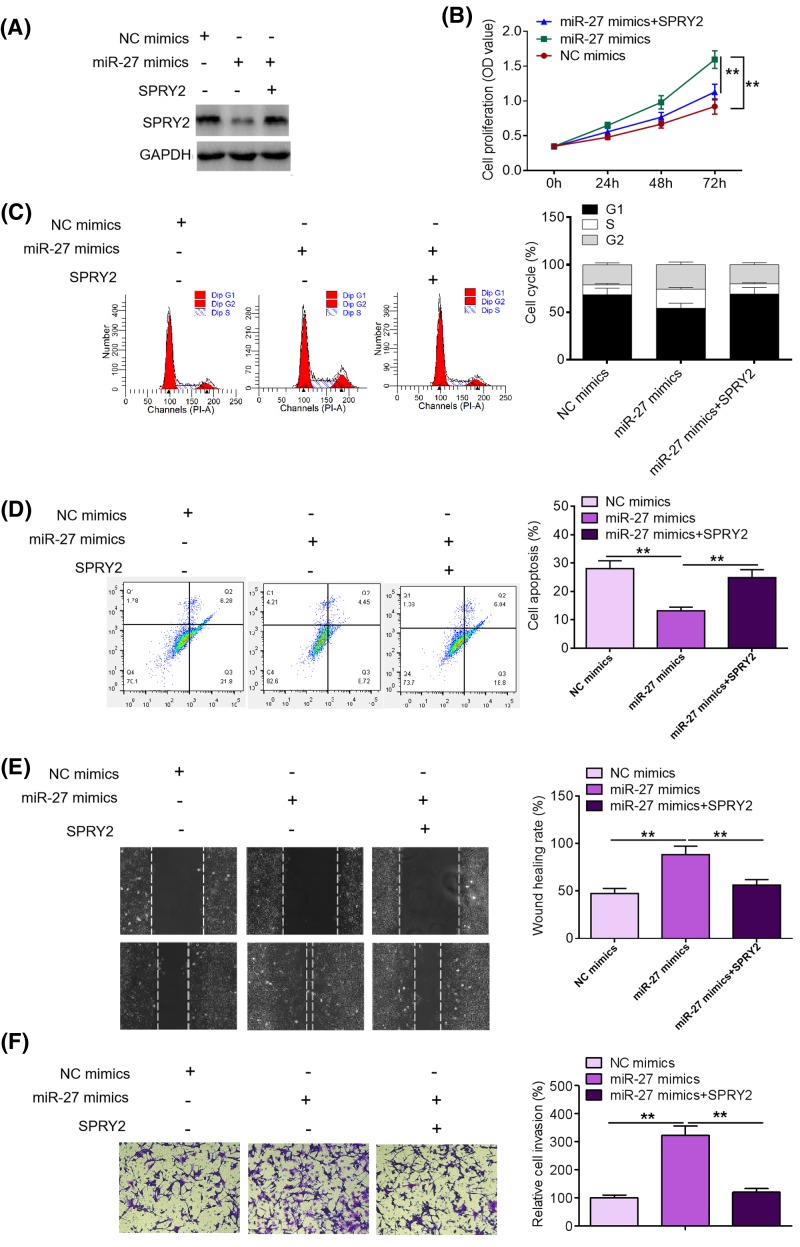
Rescue of SPRY2 expression reverses the promoting effects of miR-27 on MM cell proliferation and invasion (**A**) SPRY2 protein expression was determined by Western blotting after rescue of SPRY2 expression in miR-27 mimics-treated U266 cells. (**B**) Proliferation was evaluated by MTT assays after rescue of SPRY2 expression in miR-27 mimics-treated U266 cells. (**C**) Cell cycle was examined by FCM after rescue of SPRY2 expression in miR-27 mimics-treated U266 cells. (**D**) Cell apoptosis was evaluated by FCM analysis after rescue of SPRY2 expression in miR-27 mimics-treated U266 cells. (**E**) Migration was examined by Transwell migration assays after rescue of SPRY2 expression in miR-27 mimics-treated U266 cells. (**F**) Invasion was determined by Transwell invasion assays after rescue of SPRY2 expression in miR-27 mimics-treated U266 cells. **P<0.01.

To determine the functional link between miR-27 and SPRY2 in MM, we rescued the expression of SPRY2 in miR-27 mimics-treated U266 cells (Figure 5A). As shown in Figure 5B,C, restoration of SPRY2 expression reversed the promoting effects of miR-27 mimics on U266 cell proliferation and cell cycle progression. As exhibited in Figure 5D, rescue of SPRY2 expression mitigated the inhibitory efforts of miR-27 mimics on U266 cell apoptosis. Furthermore, we found that rescue of SPRY2 expression alleviated the promoting effects of miR-27 mimics on U266 cell migration and invasion (Figure 5E,F). Taken together, our data suggest that SPRY2 mediates the promoting effects of miR-27 on MM cell proliferation, survival, and motility.

## Discussion

MM, accounting for approximately 10% of all the hematological malignancies worldwide, has posed great threats to human life and imposed tremendous pressure on public health [2,27]. Mounting evidence has demonstrated that miRNAs function as crucial regulators in the carcinogenesis and malignant progression of various types of human neoplasms [9,10]. Recent studies have shown that ectopic expression of miRNAs contributes to the initiation and development of human MM. Zhang et al. [15] demonstrated that miR-29b was significantly down-regulated in MM and that decreased miR-29b expression inhibited MM cell apoptosis. Yu et al. [17] reported that miR-497 was significantly down-regulated in MM and that miR-497 exerted its tumor-suppressing role by targetting PBX3. In spite of great advances in the diagnosis and therapy of MM in the last several decades, the long-term prognosis of patients remains quite unsatisfactory. Hence, there is a pressing demand to seek novel diagnostic biomarkers and exploit effective therapeutic strategies for MM.

Past studies have reported that miR-27 was dysregulated and implicated in multiple types of human neoplasms. Zhang et al. [11] demonstrated that miR-27 promoted gastric cancer cell metastasis by inducing epithelial-to-mesenchymal transition. Tang et al. [12] found that miR-27 was significantly up-regulated in human invasive breast cancer and that increased miR-27 expression correlated with poor prognosis of patients. Tanak et al. [13] reported that miR-27 was highly expressed in esophageal cancer and that high miR-27 expression was associated with chemoresistance. Wang et al. [14] revealed that miR-27 up-regulation promoted thyroid cancer cell migration, invasion, and angiogenesis. However, the biological role of miR-27 in human MM remains largely unknown. In the present study, we found that miR-27 was significantly up-regulated in MM tissues compared with normal bone marrow tissues of healthy donors and that increased miR-27 expression was associated with poor prognosis of MM patients. Collectively, our data suggest that miR-27 may function as an oncogene in MM.

To gain a better understanding of the role of miR-27 in MM, we subsequently performed functional investigations. In the present study, gain- and loss-of-function approaches were used to characterize the functional role of miR-27. Functional studies demonstrated that miR-27 overexpression accelerated MM cell proliferation, cell cycle progression, cell survival, and invasion, whereas miR-27 ablation suppressed MM cell proliferation, cell survival, and motility. Moreover, we noticed that miR-27 overexpression inhibited tumor growth in mouse xenograft models. To sum up, our findings indicate that miR-27 exerts oncogenic functions in MM.

To clarify the potential molecular mechanisms by which miR-27 facilitates MM cell proliferation and motility, we conducted bioinformatics analysis using TargetScan online software and selected SPRY2 as a candidate target of miR-27. Furthermore, dual luciferase reporter assays identified SPRY2 as a downstream direct target of miR-27. It is well documented that SPRY2 is an endogenous inhibitor of MAPK signaling pathway, whose activation is closely linked to uncontrolled cell proliferation and enhanced cell motility [28,29]. Besides, SPRY2 has also been identified as a crucial negative regulator of multiple tumor-driving signaling pathways, such as ERK1/2, Smad2 and EGFR pathways [30–32]. Previous studies have demonstrated that decreased SPRY2 expression contributes to the tumorigenesis and progression of liver cancer [17], prostate cancer [33], and endometrial carcinoma [34]. Furthermore, SPRY2 has been reported to serve as a tumor suppressor in MM by repressing cell proliferation, survival, and motility [23,24].

To verify the functional connection between miR-27 and SPRY2, we rescued the expression of SPRY2 in miR-27 mimics-treated U266 cells. Interestingly, we noticed that rescuing SPRY2 expression partially reversed the promoting effects of miR-27 overexpression on U266 cell proliferation and motility. Taken together, these data indicate that miR-27 exerts its oncogenic functions in MM by directly targetting SPRY2.

In conclusion, the present study for the first time demonstrated that miR-27 was significantly up-regulated in MM tissues compared with normal bone marrow tissues of healthy donors and that increased miR-27 expression predicted poor prognosis of patients. Furthermore, mechanistic studies revealed that miR-27 functioned as an oncogene in MM through negative regulation of SPRY2. Thus, miR-27/SPRY2 axis may partially uncover the molecular mechanisms underlying MM development and represent a promising therapeutic target for MM. Nonetheless, further studies are required to validate the therapeutic value of targetting miR-27/SPRY2 axis in MM.

## Abbreviations

EGFR: epidermal growth factor receptor
ERK1/2: extracellular signal-regulated kinase 1/2
MAPK: mitogen-activated protein kinase
MM: multiple myeloma
NC: negative control
NPC: normal plasma cell
qRT-PCR: quantitative real-time PCR
SPRY2: Sprouty homolog 2
